# Ultrasound-guided nusinersen administration for spinal muscular atrophy patients with severe scoliosis: an observational study

**DOI:** 10.1186/s13023-021-01903-4

**Published:** 2021-06-13

**Authors:** Jiao Zhang, Xulei Cui, Si Chen, Yi Dai, Yuguang Huang, Shuyang Zhang

**Affiliations:** 1 Anaesthesiology Department, Peking Union Medical College Hospital, Chinese Academy of Medical Sciences, and Peking Union Medical College, Shuaifuyuan 1#, Beijing, 100730 China; 2 Neurology Department, Peking Union Medical College Hospital, Chinese Academy of Medical Sciences, and Peking Union Medical College, Beijing, China; 3 Cardiology Department, Peking Union Medical College Hospital, Chinese Academy of Medical Sciences, and Peking Union Medical College, Shuaifuyuan 1#, Beijing, 100730 China

**Keywords:** Spinal muscular atrophy, Severe scoliosis, Ultrasound-guided, Lumbar puncture

## Abstract

**Background:**

This observational study describes our experience delivering nusinersen through lumbar puncture with real-time ultrasound guidance in spinal muscular atrophy (SMA) patients with severe scoliosis.

**Results:**

Intrathecal nusinersen via real-time ultrasound-guided lumbar puncture was given to three patients who had severe thoracic and lumbar scoliosis: a 34-year-old female with type 3a SMA, a 28-year-old male with type 2a SMA, and a 14-year-old girl with type 3a SMA. Lumbar puncture was performed without sedation under ultrasound guidance using a 22G echogenic needle in the interlaminar aspect of the L4–L5 or L5–S1 interspace and a full dose of nusinersen (12 mg/5 mL) was injected after visualizing free cerebrospinal fluid flow. Patients completed their four loading doses and one maintenance dose of nusinersen. All 15 procedures were successful and well tolerated.

**Conclusions:**

Real-time ultrasound-guided lumbar puncture is an effective and radiation-free technique to administer intrathecal nusinersen in SMA patients with severe scoliosis when done by practitioners with expertise in this procedure.

**Supplementary Information:**

The online version contains supplementary material available at 10.1186/s13023-021-01903-4.

## Background

Spinal muscular atrophy (SMA) is a genetic, autosomal recessive neuromuscular disease characterized by progressive muscle atrophy, early development of joint contractures, severe scoliosis, and variable bulbar and respiratory weakness [1], which has an incidence of 1 in 12,000 live births [2]. Nusinersen is an antisense oligonucleotide which can effectively improve motor function in children with SMA by promoting full-length survival motor neuron protein production through mRNA modification, and is reported as the only available treatment for SMA in many areas. Due to its inability to cross the blood–brain barrier, this drug can only be administered intrathecally through lumbar puncture. However, standard lumbar puncture can be challenging in SMA patients with progressive scoliosis, which has an incidence as high as 100% in certain subtypes (1c and 2a) [1]. Although computed tomography (CT)- or fluoroscopy-guided techniques have previously been reported to facilitate intrathecal injection of nusinersen in SMA patients with aberrant spinal anatomies [3–6], the potential health risks (e.g., cancer) from ionizing radiation should always be a concern, since repeated injections and exposures are needed [7]. The nonradiation, real-time, ultrasound-guided technique, which has been reported to be successfully used by anesthesiologists for central neuraxial block in patients with moderate to severe lumbar scoliosis [8, 9], may be a valuable alternative for nusinersen delivery in this group of patients.

In the current study, we report our clinical experience on a total of 15 repeated nusinersen deliveries through ultrasound-guided real-time lumbar puncture in three SMA patients with severe scoliosis, including detail on the technical essentials of this technique.

## Methods

### Study participants

**Fig. 1:**
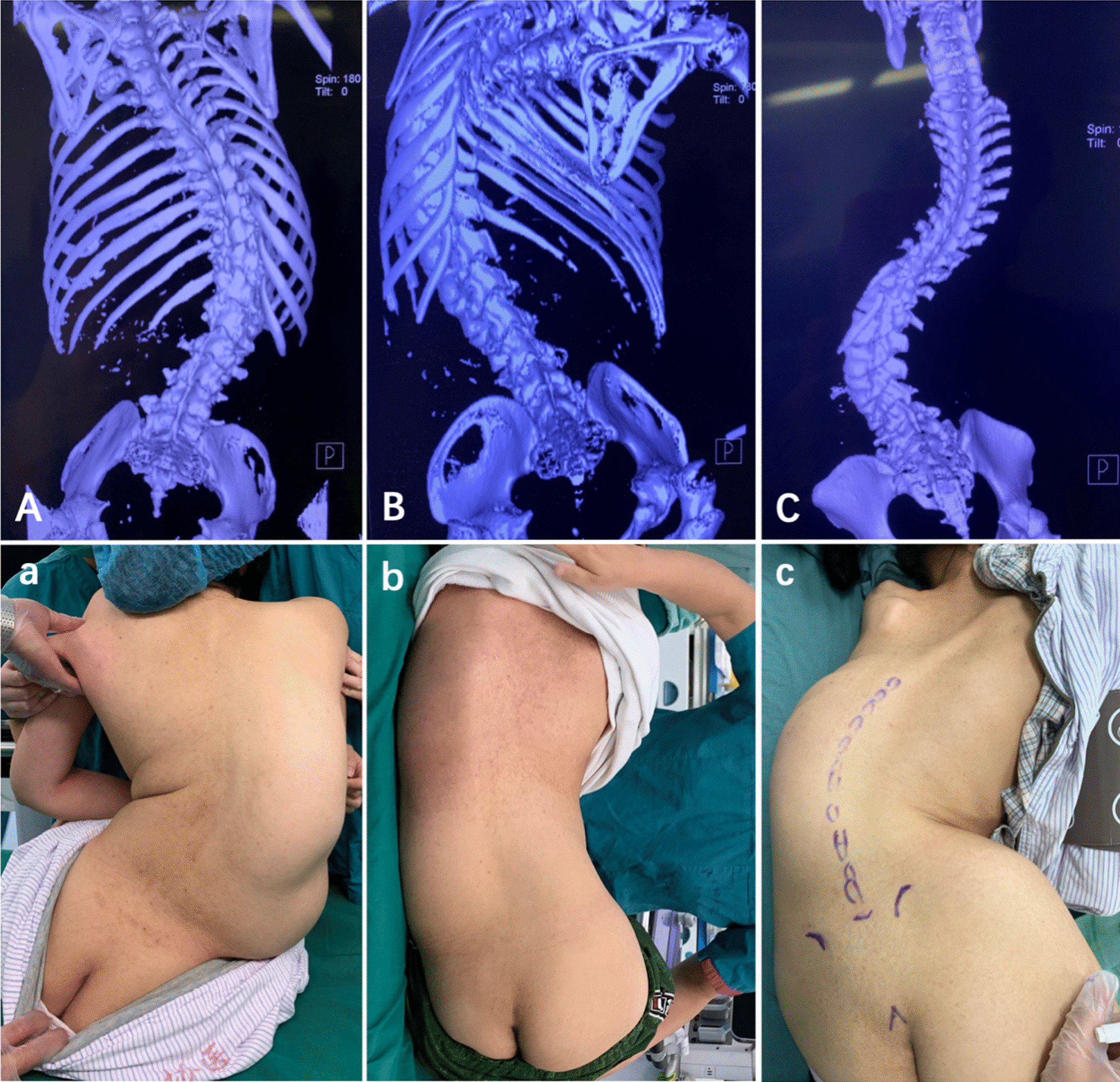
Three-dimensional CT images and appearance of the back in the three SMA patients with severe scoliosis. **A** and **a** from Patient 1, **B** and **b** from Patient 2, **C** and **c** from Patient 3

The institutional review board approved this retrospective study with a waiver for informed consent. From October 30, 2019 to July 23, 2020, patients with SMA and severe scoliosis seen by the neurology physician at our institution were referred to the anesthesiology department for intrathecal nusinersen administration because of the expected difficulty in obtaining intrathecal access (Fig. 1).

### Pre-puncture preparation

The purpose, methodology, and potential complications of the ultrasound-guided puncture procedure, and the benefits and side effects of nusinersen, were discussed with the patients and their parents prior to initiation of therapy and each procedure. Informed consent was obtained. Spinal 3D-CT reconstructions were obtained to ascertain intrathecal access at various lumbar levels and to facilitate pre-procedure planning (Fig. 1A–C).

### Ultrasound-guided lumbar puncture

**Fig. 2:**
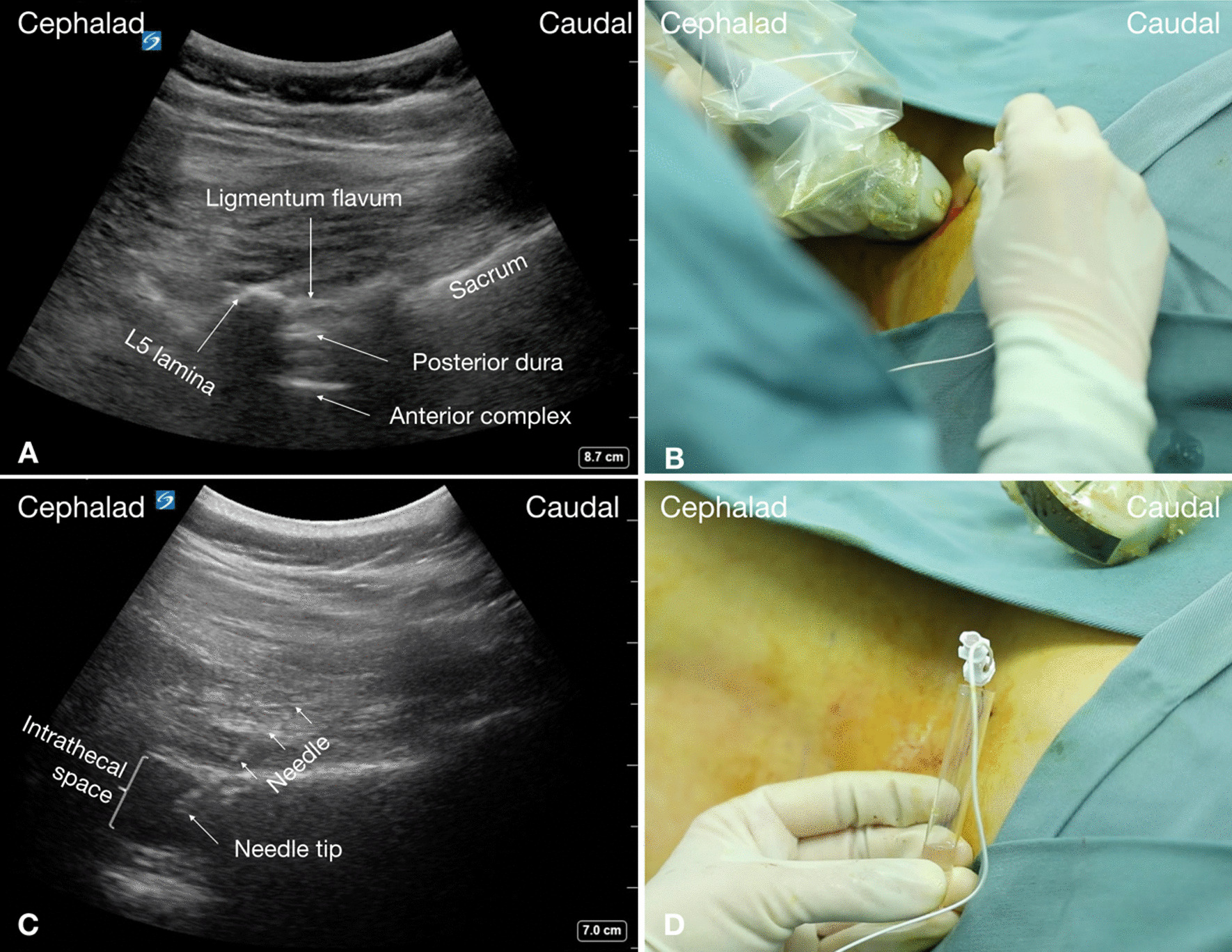
Real-time ultrasound-guided lumbar puncture in a SMA patient with severe scoliosis. **A** Paramedian sagittal oblique interlaminar sonogram of the lower lumbar spine (L5–S1) with the neuraxial structures clearly visualized through the acoustic window of the interlaminar space. **B** Ultrasound probe position of paramedian sagittal oblique scan at the level of lamina and needle orientation for lumbar puncture with in-plane approach. **C** Sonogram obtained when needle inserted into the intrathecal space. **D** Free flow of clear CSF is noted from the puncture needle

An additional movie file shows the whole process of puncture in more detail (see Additional file 1). All the punctures were performed in the operating room at the anesthesiology department. Upon arrival, standard monitoring and peripheral venous access were established for the patients. As it is important for patients to be able to communicate any concerns during the lumbar puncture procedure, sedation (intravenous midazolam) was used only when necessary to reduce the patient’s severe anxiety prior to some of the procedures. Each patient was placed in the lateral decubitus position with the knees flexed to improve lumbar space access. The patient’s head and body were supported by fitted padding. A pre-procedural ultrasound scan of the lumbar spine based on a scan protocol for scoliosis, first described by Karmakar [10], was performed using a low-frequency (8–3 MHz), curvilinear transducer (Sonosite X-Porte; Sonosite Inc., Bothell, WA, USA). Briefly, the scan followed three steps. In the first step, the positions of the spinous processes (SPs) of the lumbar spine on the patient’s back were palpated and marked using a skin-marking pen (Fig. 1C). Next, a line connecting the SPs was drawn, referred to as the “SP line”. The last step was to perform a paramedian sagittal scan by placing the transducer 1–2 cm lateral and parallel to the SP line with the transducer tilted slightly medially during the scan, so that the ultrasound beam was insonated in a paramedian sagittal oblique plane; this approach ensures that the ultrasound signal enters the spinal canal through the window of the interlaminar space (Fig. 2A and C). According to ultrasonography, the interlaminar spaces were visible with an acceptable acoustic window and returned good hyperechoic dura signals (Fig. 2A and Additional file 1), then the position of the transducer was marked using a skin-marking pen. After sterile preparation of the posterior back with iodophor and the ultrasound transducer draped in a sterile plastic sheath, 2 mL of 1% lidocaine was administered as a local anesthetic. A 10-cm, 22G, echogenic needle with a short-bevel tip (SonoLong Nanoline; Pajunk Inc., Geisingen, Germany) and stylet was advanced in-plane in a caudal-to-cephalad direction (Fig. 2B) toward the interlaminar space. The needle was traced on the ultrasound screen in real time (Fig. 2C). If dural puncture was not achieved after three needle passes (defined as forward advancement of the needle, i.e., needle redirection without exiting the skin, including the first pass), the operator had options to change to an alternative interlaminar space level. The exact localization of the needle tip (penetrating the dura) was confirmed on ultrasound image and by free flow of clear cerebrospinal fluid (CSF) after needle stylet removal (Fig. 2C, D, and Additional file 1). After extraction of 5 mL of CSF, 12 mg (12 mg/5 mL) of nusinersen was administrated through the needle. Subsequently, the needle was removed, and patients were placed in a comfortable position for 1 h of observation.

### Data collection

Patient characteristics including age, sex, genotype, subtype of SMA, personal history, clinical signs and symptoms, and results of CSF examination were obtained from the electronic medical records. Details of punctures (including the number of interspace level adjustments and insertion attempts per procedure) were recorded. Adverse events including, but not limited to, pain or discomfort leading to need for sedation, puncture of visceral organs, postlumbar puncture syndrome, respiratory disturbance, bleeding, and spinal ischemia were also obtained.

## Results

Real-time ultrasound-guided intrathecal injections of nusinersen were performed in three SMA patients with severe scoliosis in the anesthesiology department at the Peking Union Medical College Hospital between October 2019 and July 2020. Clinical characteristics of the patients are described in Table 1. Two patients were diagnosed with type 3a SMA and 1 with type 2a SMA. All three patients completed the four loading doses and one maintenance dose, which accounts for a total of 15 consecutive punctures. No patients required intravenous sedation or general anesthesia. All punctures used the paramedian sagittal in-plane approach with a success rate of 100%, assuring correct delivery of the drug in all patients. Among all of the 15 procedures, 14 (91.67%) were performed successfully at the interspace level of L4–L5, one at the level of L5–S1.

**Table 1:** Demographics/clinical data of patients and details of lumbar punctures

Clinical characteristics	Patient 1	Patient 2	Patient 3
Age	34	28	14
Sex	Female	Male	Female
Copies of SMN2 gene	3	3	3
SMA subtype	3a	2a	3a
Delivery mode	Natural labor	Natural labor	Natural labor
Cobb angle (°)	106	130	103
Ambulation	Wheelchair-bound	Wheelchair-bound	Travel short distances with assistance
Positive signs	Symmetric severe muscle weakness and wasting; tongue atrophy with fasciculations; areflexia; quadriparesis; joint contractures and ankylosis of the limbs	Symmetric severe muscle weakness and wasting; tongue atrophy with fasciculations; areflexia; joint contractures and ankylosis of the limbs	Proximal weakness affecting the legs; tongue atrophy; areflexia; joint contractures and ankylosis of both feet
Procedures (no.)	5	5	5
Need for sedation per procedure (N or Y)	N/N/N/N/N	N/N/N/N/N	N/N/N/N/N
Number of interspace level adjustments per procedure	2/2/1/1/1	2/1/1/1/1	1/1/1/1/1
Number of total insertion attempts^a^ per procedure	5/3/2/2/1	3/2/2/1/1	2/2/1/1/1
Final interspace level at which lumbar injection was done per procedure	L5–S1/L4–L5/L4–L5/L4–L5/L4–L5	L4–L5/L4–L5/L4–L5/L4–L5/L4–L5	L4–L5/L4–L5/L4–L5/L4–L5/L4–L5
Operation time^b^ (min)	66/59/34/35/30	67/45/33/31/28	58/50/40/35/32
Puncture time^c^ (min)	23/15/8/6/3	16/10/9/3/3	18/12/7/4/3
Puncture distance^d^ (cm)	8.1/7.8/7.9/7.5/7.7	8.8/8.3/8.9/9.0/8.5	7.3/7.7/7.5/7.0/6.8
Pretreatment^e^ signs and symptoms	Quadriparesis; preserved hand fine motor functioning to use computer and write; mild dysarthria; able to brush her teeth	Quadriparesis; preserved hand motor functioning to use mouse but still has difficulty in typing	Preserved upper extremity functioning to feed herself, brush her teeth, use mobile phone, write, and do schoolwork
Posttreatment^f^ signs and symptoms	Louder voice; increased upper limb strength; improved endurance and head control; able to lift her left foot off the bed; once able to turn over autonomously	Louder voice; improved masticatory movement; able to play computer games; slight movement of feet	Independent kneeling position and crawl one to two steps; able to sit up on her own; caregivers reported easier to facilitate the patient with walking
Hammersmith Functional Motor Scale-Expanded score (before the first/after the last nusinersen treatment)	1/7	0/2	23/34
Adverse events^g^	No	No	Headache and nausea

*SMA* spinal muscular atrophy, *SMN* survival motor neuron

^a^Number of total insertion attempts, defined as the number of any separate skin puncture by a needle

^b^The time interval between patients arriving at, and leaving the operation room

^c^The time interval from needle insertion to cerebrospinal fluid outflow

^d^The whole needle trajectory distance beneath the skin

^e^Baseline neurologic function prior to first dose of nusinersen

^f^Neurologic function after the last treatment of nusinersen

^g^Adverse events including: headache, back pain, nausea, constipation, dizziness, upper respiratory infection

In detail, for the first puncture of Patient 1, we initially chose to inject at the level of interspace L2–L3 based on the 3D-CT image, but failed after two attempts because of the steep angle of the needle path, which was the only path available due to the curvature of the vertebral column. Following that, we changed to the level of L5–S1 based on the ultrasound image and succeeded after two attempts. The second puncture of Patient 1, and the first puncture of Patient 2, failed at the first attempts at level L5–S1, due to the inability to withdraw CSF for uncertain reasons, even though the needle tips were seen underneath the hypoechoic dorsal dura on the ultrasound image. Finally, both punctures were successful at the second attempts at level L4–L5. For all the following procedures, level L4–L5 was selected for the first attempt and all succeeded.

All procedures were technically successful and well tolerated. Minor complications included Patient 3 reporting positional headache and nausea after the second puncture, which was resolved by bed rest. No severe complications were recorded. CSF tests and laboratory tests including platelet counts, coagulation profile, and renal and liver function tests remained unremarkable in all patients.

## Discussion

In this study, we present the results of real-time ultrasound-guided lumbar intrathecal administration of nusinersen in SMA patients with severe scoliosis. The success rate was 100% with no major complications.

### Technique

Lumbar puncture is a routine practice performed by anesthesiologists. The application of ultrasound in assisting lumbar puncture can be traced back to the 1970s [11], and was first used in patients with abnormal spinal anatomy in 1999 [12]. With improvements in ultrasonic imaging quality and greater understanding of the sonoanatomy of the spine among investigators, techniques of real-time ultrasound-guided spine puncture were investigated [13–16] and reported to be successfully used in patients with challenging spinal anatomy in 2010 [17]. In the current study, we chose in all procedures to use the real-time ultrasound-guided in-plane approach, advancing the needle in the plane of the ultrasound beam, through the longitudinal paramedian sagittal oblique interlaminar view, which was first described by Karmakar [14].

During lumbar spinal sonography, the paramedian sagittal oblique interlaminar approach possesses the advantage of providing a better sonographic window into the vertebral canal from the interlaminar space compared with the interspinous space (median) approach [18, 19], and is more successful in patients with a difficult anatomy [20, 21]. Performers of the technique should be familiar with the sonoanatomy of the interlaminar, the intrathecal, and the surrounding bony structures. Sonographically, the interlaminar space is the gap between the adjoining lamina and is the “acoustic window” through which the neuraxial structures are visualized within the spinal canal (Fig. 2A). The ligamentum flavum appears as a hyperechoic band across adjacent lamina (Fig. 2A). The posterior dura is the next hyperechoic structure anterior to the ligamentum flavum (Fig. 2A), and the epidural space between the ligamentum flavum and the posterior dura is hypoechoic. The ligamentum flavum and the posterior dura may also be seen as a single linear hyperechoic structure, which is referred to as the “posterior complex”. The intrathecal space with the CSF is the anechoic space anterior to the posterior dura. The deeper linear hyperechoic structure is the “anterior complex” composed of the anterior dura, the posterior longitudinal ligament, and the posterior surface of the vertebral body, which are of similar echogenicity and closely opposed to each other. To accurately obtain this paramedian sagittal oblique interlaminar window in SMA patients with complex deformity of the spine, we recommend to strictly follow the currently used three-step scan protocol [10].

The key to the real-time ultrasound-guided in-plane technique used in this study is in obtaining and maintaining alignment of the ultrasound beam with the needle and the acoustic window into the interlaminar space during the whole procedure, which is crucial to guarantee the success of lumbar puncture [16]. Advanced skills in hand–eye coordination and needle-transducer manipulation will definitely be required to successfully perform this technique [16]. In the current study, there was no efflux of CSF in two punctures, though the hypoechoic “needle tips” were seen in the intrathecal space on the ultrasound image. We speculate that imprecise alignment of the needle along the ultrasound beam might have been responsible. This means that the hypoechoic dots we saw on the ultrasound image may actually represent the transverse section of the needle body instead, and the real needle tip had been advanced too deep into the anterior epidural space. Additionally, factors such as muscle atrophy, muscle weakness, and severe scoliosis in this group of patients, which may lead to CSF reflux or uneven CSF pressure in the subarachnoid space, can also contribute to poor CSF outflow. There were another two failed attempts (in Patients 1 and 2) in which the intrathecal structures were clear in the ultrasound image, but we were unable to insert the needle through the interlaminar space. This may have been due to the steep needle-puncturing angle. A steeper needle-puncturing angle will inevitably attenuate the imaging quality of the needle and make it difficult to manipulate the needle based on the ultrasound image. Therefore, when determining the optimal puncture level during the pre-procedure scan, both the imaging quality of the given interlaminar space and the predicted needle-puncturing angle should be considered.

### Safety

In terms of safety, a prominent advantage of the current technique is its inherent radiation-free property when compared with traditionally used fluoroscopy- or CT-guided techniques. As therapy with nusinersen is long term, the cumulative radiation exposure from repeated puncture may be of concern because of its deleterious effects for human health in inducing cancers (e.g., colon cancer, lung cancer, and leukemia) [4, 7]. Additionally, by using the real-time ultrasound-guided technique, advancement of the needle could be visualized during the whole procedure, which is particularly helpful in avoiding unnecessary vascular or visceral injuries.

Here, we successfully and safely administered nusinersen intrathecally in all 15 procedures. No severe adverse events were noted, except for one patient reporting positional headache and nausea after the second injection, which was resolved by bed rest. The overall incidence of postdural-puncture headache (PDPH) in this study was 8.3% (1/15), which is higher than the rate of 2.0% previously reported by Kathryn DelPizzo et al. in a prospective study including 300 15–45-year-old patients receiving combined spinal-epidural anesthesia or spinal anesthesia with a 27G pencil-tip needle [22]. One possible explanation might be the 22G short-beveled needle, a kind of traumatic needle, that we chose to use in this study. This was the only echogenic needle available in our medical center. As an echogenic property is essential for improving the ultrasound image of the needle body and therefore the success rate of the puncture, this characteristic is the first consideration when we choose the needle type. However, we believe an atraumatic echogenic needle should always be the best choice if it is available. Even so, it is of note that the incidence of PDPH in the current study might not be representative due to the limited number of patients and punctures; further study with a larger sample is needed.

### Limitations

The current study has several limitations. Firstly, this is a retrospective and nonblinded evaluation of a technique used in only a few patients with a limited number of punctures, and no control groups were utilized to compare CT guidance to the ultrasound technique in our study. Secondly, our experience is mainly based on patients with severe scoliosis but no previous spinal surgery (i.e., rod placement or fusion); such cases may present greater challenges for lumbar puncture since the interlaminar space might not be visible under ultrasound guidance. Even so, ultrasound assistance or a real-time guidance technique might also be valuable in SMA patients with spinal fusion instrumentation, since similar procedures have been successfully applied in other groups of patients to facilitate interlaminar and transforaminal lumbar puncture [10].

## Conclusion

The real-time ultrasound-guided technique can be successfully used to assist lumbar nusinersen administration in SMA patients with severe scoliosis. The advantage of the current technique is that it is free of exposure to radiation, provides real-time images, and improves patient comfort during the whole procedure. A sound knowledge of the sonoanatomy of the spinal deformity and skilled manipulation techniques are needed for practitioners to apply this technique safely and effectively.

## Supplementary Information

**Additional file 1** The operation video shows the whole process of the puncture.

## Data Availability

All study data, including raw and analyzed data and materials, will be available from the corresponding author on reasonable request.
